# STAT4 is expressed in neutrophils and promotes antimicrobial immunity

**DOI:** 10.1172/jci.insight.141326

**Published:** 2021-07-22

**Authors:** Pegah Mehrpouya-Bahrami, Alina K. Moriarty, Paulo De Melo, W. Coles Keeter, Nada S. Alakhras, Andrew S. Nelson, Madeline Hoover, Maria S. Barrios, Jerry L. Nadler, C. Henrique Serezani, Mark H. Kaplan, Elena V. Galkina

**Affiliations:** 1 Department of Microbiology and Immunology and; 2 Herman B Wells Center for Pediatric Research, Department of Pediatrics, School of Medicine, Indiana University, Indianapolis, Indiana, USA.; 3 Department of Microbiology and Molecular Cell Biology, Eastern Virginia Medical School, Norfolk, Virginia, USA.; 4 Division of Infectious Diseases, Department of Medicine, Vanderbilt University Medical Center, Nashville, Tennessee, USA.; 5 Department of Biochemistry and Molecular Biology, School of Medicine, Indiana University, Indianapolis, Indiana, USA.; 6 Departments of Medicine and Pharmacology, School of Medicine, New York Medical College, Valhalla, New York, USA.; 7 Department of Pathology, Microbiology and Immunology, Vanderbilt University Medical Center, Nashville, Tennessee, USA.

**Keywords:** Immunology, Neutrophils

## Abstract

Signal transducer and activator of transcription 4 (STAT4) is expressed in hematopoietic cells and plays a key role in the differentiation of T helper 1 cells. Although STAT4 is required for immunity to intracellular pathogens, the T cell–independent protective mechanisms of STAT4 are not clearly defined. In this report, we demonstrate that STAT4-deficient mice were acutely sensitive to methicillin-resistant *Staphylococcus aureus* (MRSA) infection. We show that STAT4 was expressed in neutrophils and activated by IL-12 via a JAK2-dependent pathway. We demonstrate that STAT4 was required for multiple neutrophil functions, including IL-12–induced ROS production, chemotaxis, and production of the neutrophil extracellular traps. Importantly, myeloid-specific and neutrophil-specific deletion of STAT4 resulted in enhanced susceptibility to MRSA, demonstrating the key role of STAT4 in the in vivo function of these cells. Thus, these studies identify STAT4 as an essential regulator of neutrophil functions and a component of innate immune responses in vivo.

## Graphical abstract

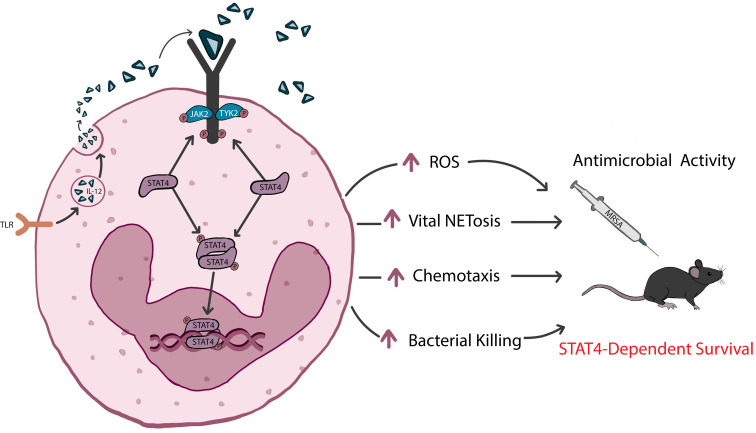

## Introduction

Signal transducer and activator of transcription (STAT) proteins are a family of factors implicated in various biological processes, including the induction of genes involved in cell differentiation (1). STAT4, activated downstream of IL-12, is the only STAT that shows tissue-restricted expression, with mRNA constitutively expressed in lymphoid cells and inducibly expressed in monocytes and macrophages (2–4). STAT4 is required for all known IL-12 biological functions, including the induction of IFN-γ and the promotion of T helper type 1 (Th1) differentiation (5, 6). Single nucleotide polymorphisms (SNPs) of the STAT4 gene have been associated with asthma, Sjögren’s syndrome, rheumatoid arthritis, and systemic lupus erythematosus (7). In models of infection and autoimmunity, STAT4 is a critical component in developing inflammation (8). STAT4-deficient mice are susceptible to infection with intracellular pathogens, have decreased delayed-type hypersensitivity (DTH) responses (8), and have attenuated T cell responses (5, 6). In contrast, STAT4-deficient mice are refractory to the induction of inflammatory conditions, including colitis, arthritis, diabetes, myocarditis, and experimental autoimmune encephalitis (7). Thus, STAT4 is required for inflammatory immunity.

Many of the studies on STAT4 function have focused on T cells. Only a few reports have investigated STAT4’s role in macrophages and dendritic cells. The homeostatic numbers of myeloid cells are normal in the absence of STAT4, despite alterations in hematopoietic progenitor cell numbers (9). In human monocytes and mouse dendritic cells, STAT4 expression is induced with maturation, and rheumatoid synovia-isolated macrophages express STAT4 (3, 4). Macrophage stimulation with either IL-12 or type I IFNs results in STAT4-dependent expression of *Ifng*, *Tnfa*, and *Nos2* (3, 4, 10, 11). Thus, STAT4 is clearly functional in monocytes and macrophages. While STAT4 is involved in shaping inflammatory responses, it has not been defined whether STAT4 is expressed in neutrophils or whether its activation would affect neutrophil functions in normal and pathological conditions.

Notably, there is growing evidence that STAT4 is functionally important in innate cells. SNPs in the STAT4 gene are associated with diseases with a clear link to innate immunity (7, 8). Moreover, patients with mutations in IL-12 signaling become susceptible to bacterial infections (12, 13). Infection of *Stat4*^–/–^ mice with *Salmonella typhimurium*, *Klebsiella pneumonia*, and *Mycobacterium tuberculosis* results in a bacterial burden that is orders of magnitude greater than controls (8). Godshall and colleagues demonstrated that STAT4 is required for antibacterial defense during polymicrobial peritonitis in the cecal ligation and puncture model (14). Interestingly, STAT4 deficiency is also linked to impaired production of proinflammatory cytokines in inflammation induced by *Pseudomonas aeruginosa* (*P*. *aeruginosa*) in BALB/c mice but was not implicated in bacterial clearance (15). STAT4 expression in innate lymphoid cells contributes to *Listeria* *monocytogenes* immunity (16). Overall, while there were some indications suggesting the involvement of the IL-12/STAT4 pathway in the host defense, the cells responsible for STAT4-dependent antibacterial immunity and the cellular processes that control antibacterial responses have not been identified. In this report, using STAT4–conditional mutant mice and bacterial infection in vivo, we define STAT4-dependent neutrophil functions during infection.

## Results

### STAT4 is expressed in neutrophils, is activated by IL-12, and regulates gene expression.

Much of the research on IL-12– and IFN-α–dependent functions of STAT4 has been performed in T and NK cells (8); however, evidence suggests that STAT4 is also expressed in activated monocytes, macrophages, and dendritic cells. Additionally, an essential role of the STAT4 pathway has been demonstrated in both the cecal ligation and puncture model (14) and *P*. *aeruginosa*–induced inflammation (15). The rapidity of the response observed during infections suggested that STAT4 function in innate immune cells was required for this response, but specific subpopulations of STAT4-dependent innate immune cells that are responsible for the phenotype have not been identified.

**Figure 1:**
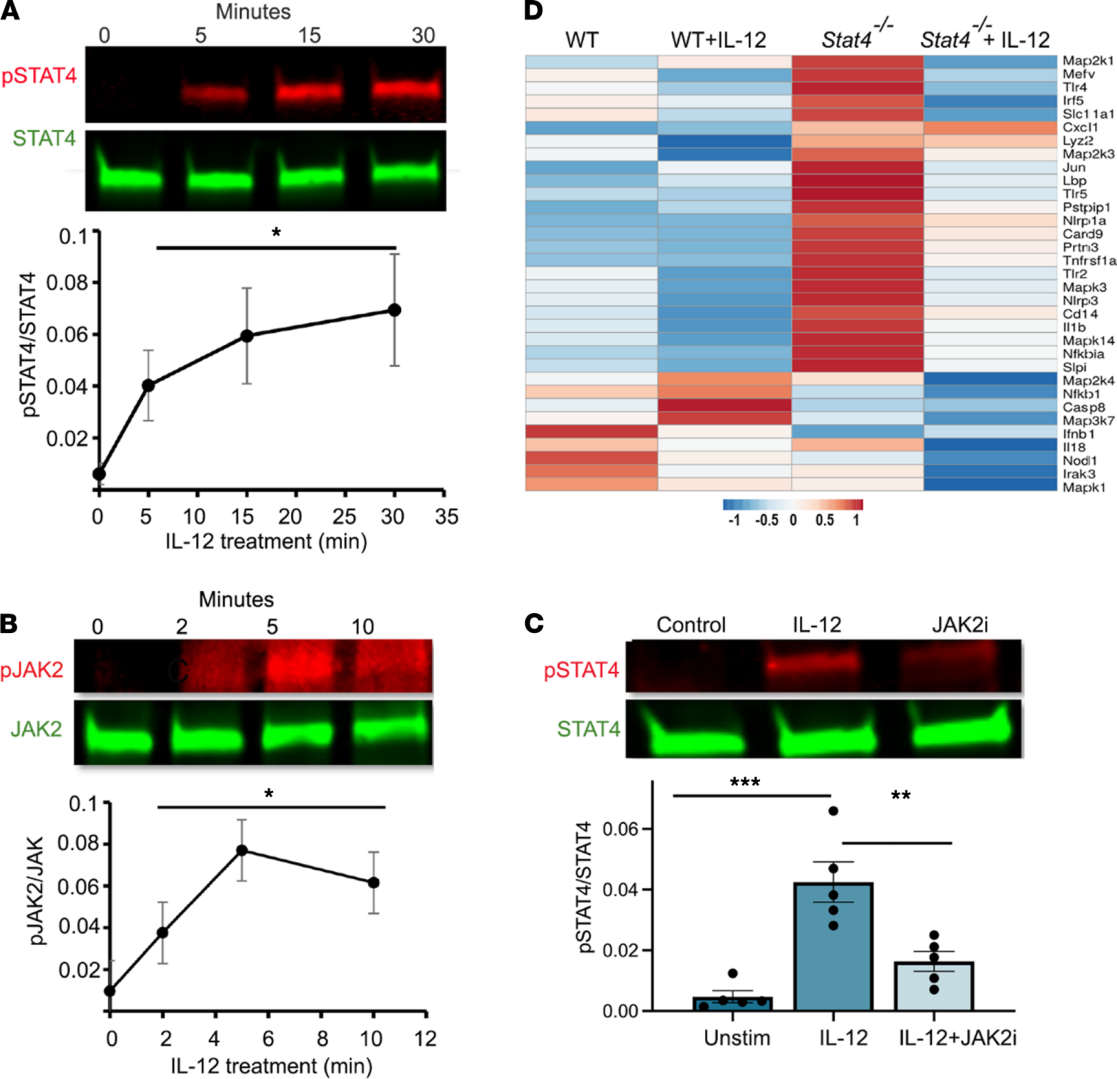
STAT4 is expressed in neutrophils, is activated by IL-12, and regulates gene expression profile of neutrophils. BM neutrophils were treated with IL-12 (40 ng/mL) for indicated time points. (**A**) The expression of phosphorylated STAT4/STAT4 (p-STAT4/STAT4) or (**B**) p-JAK/JAK was examined by Western blot. Representative image from 3 independent experiments (*n* = 3–5 mice total). (**C**) BM neutrophils were either pretreated with the JAK2 inhibitor (JAK2i) gandotinib (1 μM) or left untreated, and 15 minutes later, all samples were stimulated with IL-12 for 30 minutes, and expression of p-STAT4/STAT4 was determined by Western blotting at the indicated time points (*n* = 5 mice/per group in 3 independent experiments). (**D**) Heatmaps of real-time RT^2^ Profiler PCR Array showing differential expression of genes between WT and *Stat4^–/–^* IL-12–treated and –untreated samples. Differences between groups were assessed by 2-tailed Student’s *t* test (*P < 0.05), using the RT^2^ Profiler PCR Array Analysis (*n* = 3 samples/group). Data are mean ± SEM; *P < 0.05, **P < 0.01, ***P < 0.001 using 1-way ANOVA followed by Tukey-Kramer post hoc test.

Neutrophils are a critical early component of innate immunity and play a central role in defense against various bacterial pathogens, such as *S*. *aureus* (17). To date, no reports demonstrate STAT4 expression or function in neutrophils. Thus, we explored whether neutrophils express STAT4 and respond to IL-12, a key STAT4-activating cytokine (8). Murine neutrophils isolated from the bone marrow (BM) of WT mice clearly expressed STAT4 (Figure 1A). Notably, IL-12 treatment induced time-dependent phosphorylation of STAT4 in BM neutrophils (Figure 1A). To further understand how IL-12 induces STAT4 phosphorylation in neutrophils, we next focused on intracellular signal transduction processes initiated by type I cytokines that utilize various JAK proteins to activate STAT4 (18). We demonstrated that JAK2, an upstream signaling kinase of STAT4, underwent rapid phosphorylation upon IL-12 stimulation (Figure 1B). To confirm that IL-12 induces JAK2-dependent activation of STAT4, we used gandotinib (LY2784544), a potent, selective small-molecule JAK2 inhibitor that has dose-dependent selectivity for the JAK2 (19). As shown in Figure 1C, the treatment of IL-12–stimulated neutrophils with gandotinib diminished STAT4 phosphorylation, suggesting that IL-12 signals through the canonical JAK2/STAT4 pathway in neutrophils.

We next utilized PCR arrays that profile 84 genes related to antimicrobial response to investigate the transcriptional outcomes of STAT4 functionality in IL-12–induced neutrophil activation. The major effect of IL-12 treatment for WT neutrophils was in the upregulation of signal transduction pathways associated with inflammation, apoptosis, and cytokine signaling (*Il6*, *Ripk1*, *Tirap*, *Ticam1*), with the main effect on *Jun* expression. Unexpectedly, STAT4 deficiency in neutrophils resulted in upregulation of some genes responsible for the induction of inflammation, such as *Lbp* and *Tlr2*, *4*, and *5* (TLR activation); *Card9* and *Tnfrsf1a* (positive regulation of apoptosis, NF-κB, and ROS production); *Lyz2* and *Prtn3a* (granule formation/release); *Pstpip1* and *Mefv* (cytoskeleton and autophagy, respectively); *Jun* and *Mapk2k3*, *Mapk14*, and *Mapk3* (signal transduction); and *Nlrp1a* and *Il1b* (inflammasome formation) at the basal state (Figure 1D). In contrast, genes related to TLR-dependent regulation of innate immunity (*Nod1*, *Infb1*) were diminished in *Stat4^–/–^* versus WT neutrophils (Figure 1D). Further comparison of IL-12–treated *Stat4^–/–^* and vehicle-treated control *Stat4^–/–^* neutrophils showed that IL-12 also had additional STAT4-independent effects via regulation of inflammatory pathways (*Ifnb1*; *Irak3*; *Irf5*; *Nod1*; *Mapk1*, *3*, and *14*; and *Mefv*). Interestingly, a comparison of IL-12–activated *Stat4^–/–^* and WT neutrophils demonstrated differences in the induction of key proinflammatory genes (*Casp8*, *Map3k7*, *Mapk1*, *Nfkb1*, and *Il18*) responsible for NF-κB and inflammasome pathway activation (20). Unexpectedly, IL-12–treated *Stat4^–/–^* neutrophils also demonstrated upregulation of *Cxcl1*, *Card9*, *Lyz2*, *Nlrp1a*, *Prtn3*, *Sipi*, and *Tnfrsf1a* expression compared with IL-12–treated WT neutrophils (Figure 1D), suggesting a hyperactivated phenotype of *Stat4^–/–^* neutrophils. These data support the conclusion that IL-12/STAT4 signaling is active in neutrophils and reveal a complex role of the IL-12/STAT4 axis in the regulation of proinflammatory gene expression.

### STAT4 supports chemotaxis, migration, and IL-12–dependent ROS production and bacterial killing in neutrophils.

To avoid the complications of examining myeloid cell function in *Stat4* germline mutant mice, we generated a *Stat4^fl/fl^* mouse model and crossed to either myeloid cell–specific (*LysM^cre^*) or neutrophil-specific (*S100A8^cre^*) Cre-transgenic mice (Supplemental Figure 1, A and B; supplemental material available online with this article; https://doi.org/10.1172/jci.insight.141326DS1). Deletion of STAT4 was observed in neutrophils and macrophages in *Stat4^fl/fl^* *LysM^cre^* mice, while only neutrophils from *Stat4^fl/fl^* *S100A8^cre^* mice were deficient in STAT4 expression (Supplemental Figure 1C). Importantly, *Stat4^fl/fl^* *LysM^cre^* mice had normal development of myeloid cells in secondary lymphoid tissues and blood, and Th1 differentiation was normal in T cell cultures from *Stat4^fl/fl^* *LysM^cre^* mice (Supplemental Figure 1, D–F). In line with specific STAT4 deletion in myeloid cells, we observed diminished activation of STAT4 in methicillin-resistant *Staphylococcus aureus*– (MRSA-activated) and IL-12–activated BM-derived macrophages (BMDMs) and reduced induction of known STAT4 target genes in cultured BMDMs (Supplemental Figure 1, G and H).

**Figure 2:**
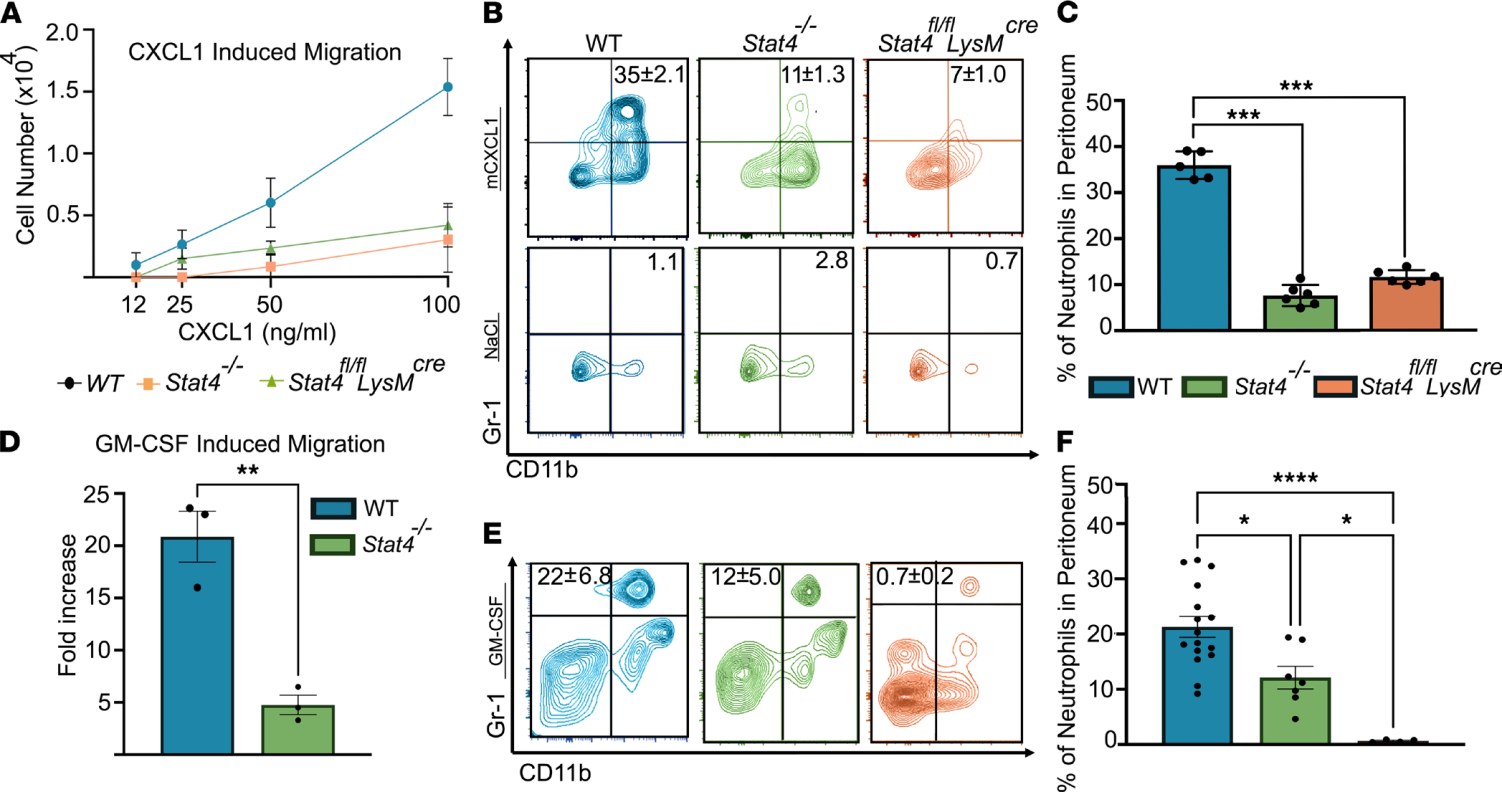
STAT4 supports chemotaxis and migration of neutrophils. (**A**) CXCL1-induced chemotaxis of WT, *Stat4^–/–^*, and *Stat4^fl/fl^* *LysM^cre^* neutrophils in a Transwell assay (*n* = 3/group in 2 independent experiments). (**B** and **C**) WT, *Stat4^–/–^*, and *Stat4^fl/fl^* *LysM^cre^* mice were injected i.p. with CXCL1 or 0.9% NaCl. After 4 hours, percentage of extravasated neutrophils in the lavage were determined (*n* = 5–6/group in 4 independent experiments). ***P < 0.001 by 1-way ANOVA followed by post hoc test. (**D**) GM-CSF–induced chemotaxis of WT and *Stat4^–/–^* neutrophils in a Transwell assay (*n* = 3/group in 3 independent experiments). *P < 0.05 using paired 2-tailed Student’s *t* test. (**E** and **F**) WT, *Stat4^–/–^*, and *Stat4^fl/fl^* *LysM^cre^* mice were injected i.p. with recombinant murine GM-CSF (rmGM-CSF), and 4 hours later percentage of extravasated neutrophils in the lavage was determined (*n* = 5–17/group in 7 independent experiments). ***P < 0.001 using Welch’s 1-way ANOVA test with Dunnett’s post hoc test.

Because neutrophils are the first responders to fight infections, these cells must migrate efficiently to the site of infection to eliminate invading pathogens (17). To test how STAT4 deficiency affects neutrophil recruitment to sites of inflammation, we first examined chemotaxis of neutrophils to CXCL1 in Transwell assays. CXCL1-induced chemotaxis was substantially decreased for neutrophils isolated from *Stat4^–/–^* and *Stat4^fl/fl^* *LysM^cre^* mice (Figure 2, A–C). We next investigated how STAT4 deletion affects neutrophil recruitment in vivo. We injected CXCL1 or saline into the peritoneal cavity (PC) of WT, *Stat4^–/–^*, and *Stat4^fl/fl^* *LysM^cre^* mice. After 4 hours, we observed greatly diminished neutrophil migration into the PC of *Stat4^–/–^* and *Stat4^fl/fl^* *LysM^cre^* compared with WT mice (Figure 2, B and C), indicating that STAT4 promotes neutrophil recruitment. GM-CSF regulates neutrophil recruitment in vivo (21). To further test the role of STAT4 in the regulation of chemotaxis, we performed GM-CSF–induced transmigration assays. We detected an approximately 4-fold decrease in migration between *Stat4^–/–^* and WT neutrophils (Figure 2D). Importantly, these data were further confirmed by results from GM-CSF–induced recruitment of neutrophils into the PC in vivo (Figure 2, E and F).

**Figure 3:**
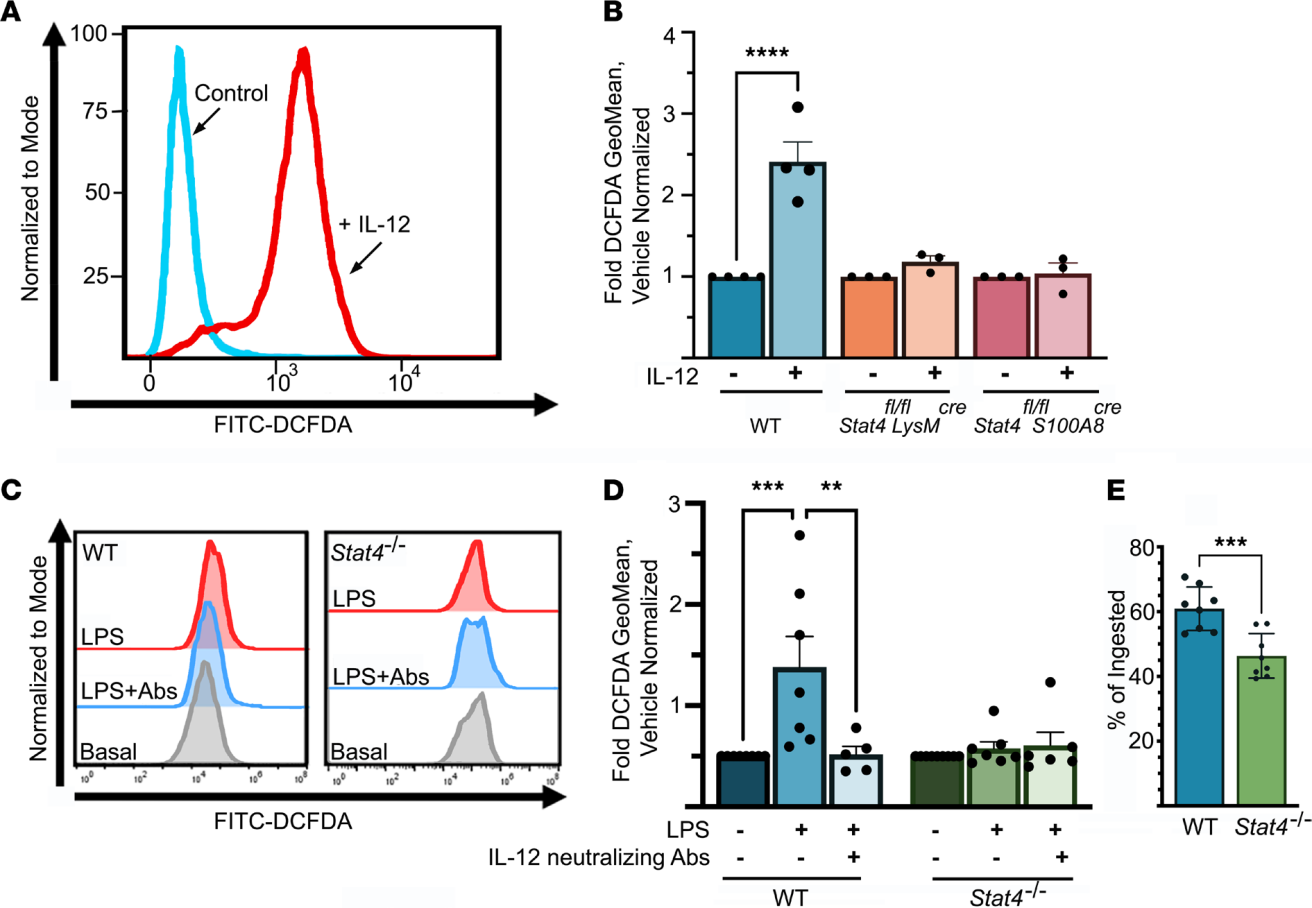
Neutrophil ROS production and bacterial killing are STAT4 dependent. (**A** and **B**) WT, *Stat4^–/–^*, and *Stat4^fl/fl^* *LysM^cre^* BM leukocytes were labeled with FITC-DCFDA, stimulated with IL-12 (40 ng/mL), stained with anti-CD11b, anti-Ly6G Abs, and analyzed for ROS production by FACS 14 hours later (*n* = 3–4/group in 2 independent experiments). FITC-DCFDA was used to measure ROS levels. (**C** and **D**) FITC-DCFDA–labeled WT, *Stat4^–/–^*, and *Stat4^fl/fl^* *LysM^cre^* BM leukocytes were either pretreated with IL-12 blocking Abs or untreated, stimulated with LPS (100 ng/mL), and analyzed for ROS production by FACS 1 hour later (*n* = 4–5/group in 2 independent experiments). (**A**–**D**) ***P < 0.001 using 1-way ANOVA followed by Tukey-Kramer post hoc test. (**E**) Bacterial killing of GFP-tagged MRSA by neutrophils from WT and *Stat4^–/–^* mice as described in *Phagocytosis and killing assays*. **P < 0.01, ***P < 0.001, ****P < 0.0001 using paired 2-tailed Student’s *t* test.

We then asked whether additional functions of neutrophils were dependent on the IL-12/STAT4 axis. As shown in Figure 3A, IL-12 induced substantial ROS production in neutrophils. In contrast, ROS production was almost abolished in *Stat4^fl/fl^* *LysM^cre^* or *Stat4^fl/fl^* *S100A8^cre^* neutrophils (Figure 3B). IL-12–induced ROS production was not due to increased apoptosis (Supplemental Figure 2, A and B). While IL-12 is an essential component that initiates STAT4 activation, we also sought to test whether other stimuli might induce STAT4-dependent activation of neutrophils. We, therefore, tested a role of STAT4 in TLR-dependent responses. Interestingly, LPS-induced ROS production was also at least partially STAT4 dependent (Figure 3, C and D). Neutrophils are capable of secreting a broad spectrum of cytokines and chemokines. Evidence demonstrates that neutrophils contain cytokines, particularly IL-12 and IL-6, in preformed granules and can quickly release these intracellular stores upon stimulation (22). Therefore, we sought to explore whether TLR-dependent neutrophil activation induces IL-12 release by neutrophils, activation of STAT4, and a subsequent increase in ROS production. The presence of neutralizing Abs to IL-12 abolished effects of LPS stimulation and ROS production and mimicked the response seen with STAT4-deficient neutrophils (Figure 3, C and D). These results suggest that TLR-induced ROS production occurs at least partially via an IL-12/STAT4–dependent pathway.

The neutrophil is equipped with several pathways for bacterial killing, including the generation of ROS and release of granules containing proteases and antimicrobial peptides. Because STAT4 regulated ROS production, we next tested whether STAT4 deficiency affects the bacterial killing capacity of neutrophils using MRSA (Figure 3E). As shown in Figure 3E, STAT4-deficient neutrophils demonstrated attenuated bacterial killing of MRSA in comparison with WT neutrophils. Phagocytosis is one of the major functions of neutrophils and the main defense mechanism against bacterial challenges. Interestingly, we found that phagocytosis of MRSA or *E*. *coli* (Supplemental Figure 2, C and D) by neutrophils was not altered in the absence of STAT4, suggesting that STAT4 modulates pathways that regulate many but not all neutrophil functions.

### IL-12–dependent chemotaxis of human neutrophils.

**Figure 4:**
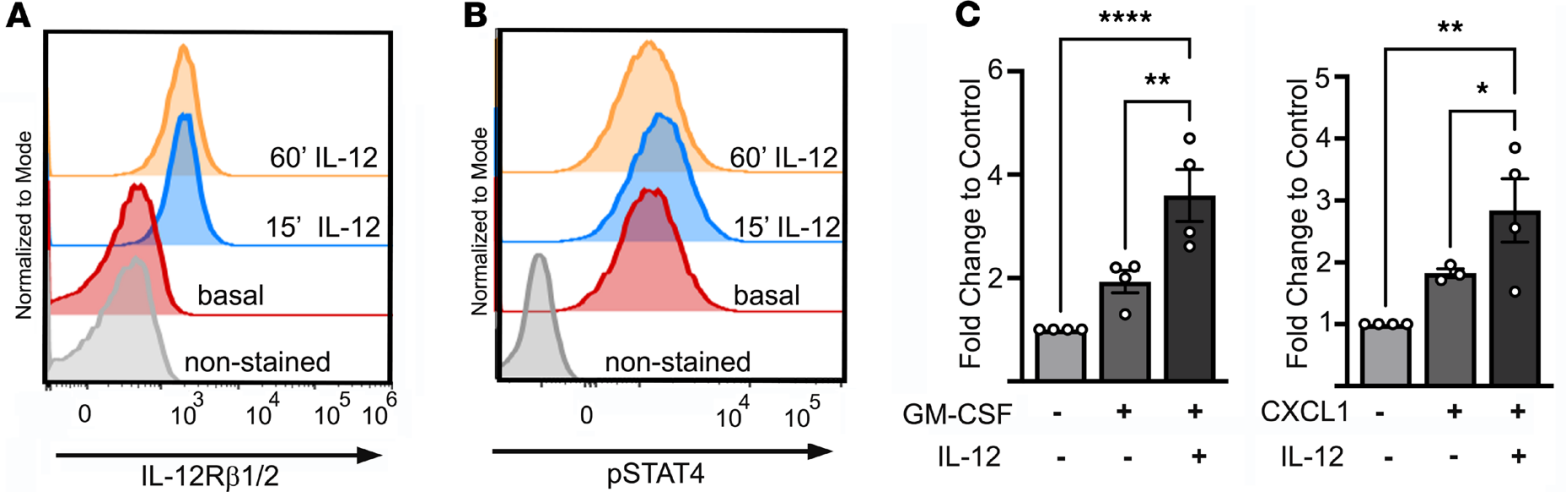
IL-12 induces STAT4 activation in human neutrophils and supports neutrophil chemotaxis in vitro. (**A** and **B**) Human neutrophils were incubated with IL-12 (40 ng/mL) for indicated time points; stained for p-STAT4–PE, IL-12Rβ1 (FITC), and IL-12Rβ2 (Alexa Fluor 488); and analyzed by FACS. Representative histogram is shown (total: 4 donors in 2 independent experiments). (**C**) Isolated human neutrophils (0.5 *×* 10^6^/200 μL in RPMI 1640+1% FBS) were either pretreated with IL-12 (40 ng/mL) or left untreated for 30 minutes and then seeded into Transwell inserts. Neutrophils migrated toward RPMI 1640 + 1% FBS media supplemented with 100 ng/mL recombinant human CXCL1 (rhCXCL1) or 50 ng/mL rGM-CSF, or migration media as a control, for 90 minutes at 37°C before being harvested and counted. The results depict neutrophil migration index normalized to the mean of the controls. *P < 0.05, **P < 0.01, ****P < 0.0001 using 1-way ANOVA followed by Tukey-Kramer post hoc test.

So far, our data demonstrated the functional activity of the IL-12 /STAT4 axis in murine neutrophils. Next, we tested the expression of IL-12Rβ1 and IL-12β2 by human peripheral blood neutrophils from healthy subjects using combined staining with anti–IL-12Rβ1-FITC and IL-12β2–Alexa Fluor 488 Abs. As shown in Figure 4A, human neutrophils expressed low but detectable levels of basal IL-12Rβ1/β2 (combined staining with anti–12Rβ1-FITC and IL-12β2–Alexa Fluor 488 is shown), which increased upon IL-12 treatment. IL-12 treatment also rapidly induced STAT4 phosphorylation in a time-dependent manner (Figure 4B). Human neutrophils not only expressed IL-12R, but they were also functionally responsive to IL-12. IL-12 increased human neutrophil chemotaxis to CXCL1 and GM-CSF in the Transwell assays, indicating that the IL-12/STAT4 axis is functional in human neutrophils (Figure 4C).

### STAT4 is required for neutrophil extracellular trap formation.

**Figure 5:**
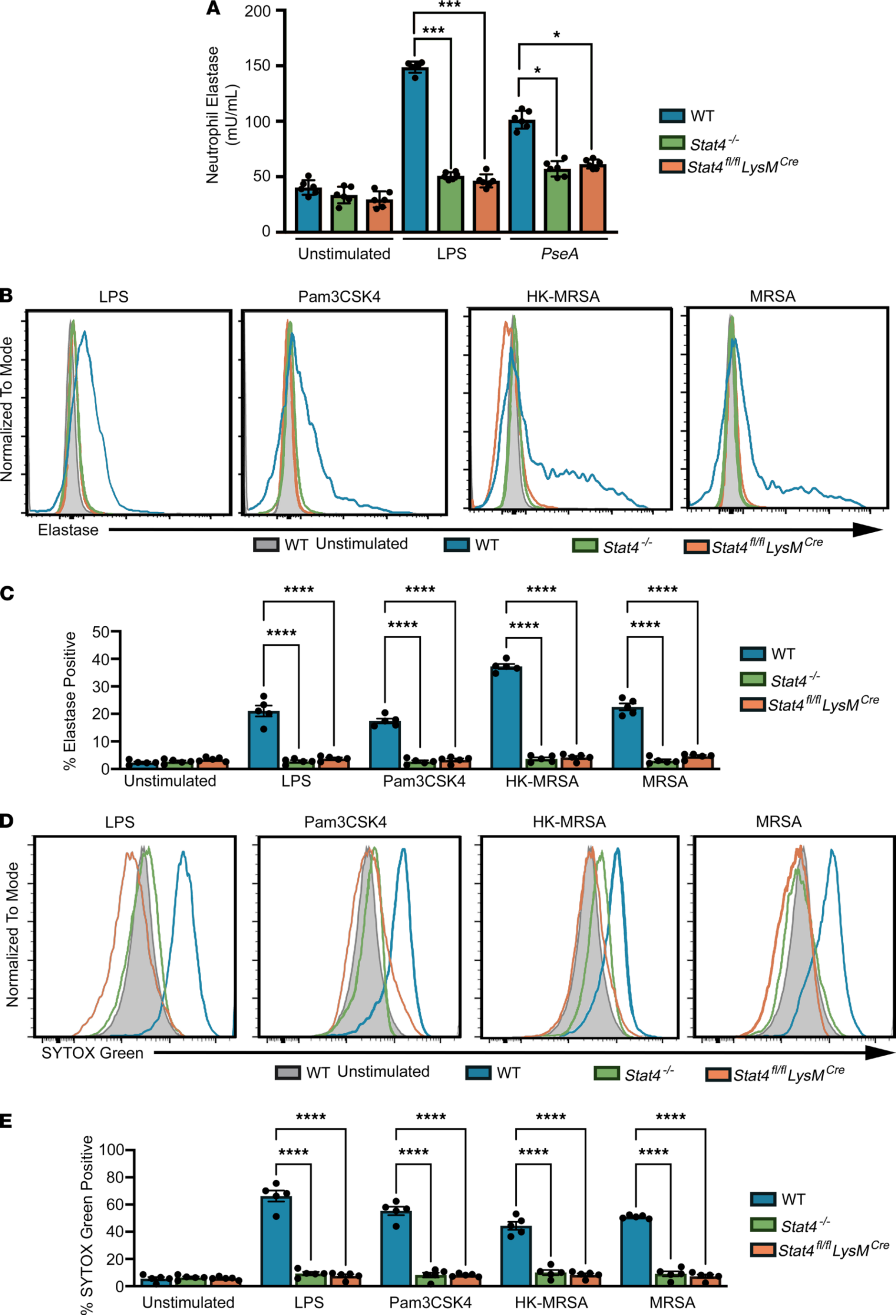
STAT4 supports TLR-dependent induction of elastase and DNA release from neutrophils. (**A**) Neutrophils from WT, *Stat4^–/–^*, and *Stat4^fl/fl^* *LysM^cre^* mice were stimulated with LPS or *P*. *aeruginosa* overnight. Supernatants were assayed for release of neutrophil elastase by ELISA (*n* = 6 mice in 2 independent experiments). (**B**) Purified neutrophils from BM cells from WT, *Stat4^–/–^*, and *Stat4^fl/fl^* *LysM^cre^* mice were stimulated with LPS (100 ng/mL), Pam3CSK4 (10 ng/mL), HK-MRSA (10:1 MOI), or MRSA (10:1 MOI) for 60 minutes; stained for elastase (**B** and **C**) or Sytox Green (**D** and **E**); and examined by flow cytometry. (**B**) Representative histogram for elastase staining. (**C**) Percentage of elastase release from total Ly6G^+^CD11b^+^ neutrophils. (**D**) Representative histogram for Sytox Green staining. (**E**) Percentage of Sytox Green–positive out of total Ly6G^+^CD11b^+^ neutrophils. (**C** and **E**) *P < 0.05, ***P < 0.001, ****P < 0.001 using 1-way ANOVA followed by Tukey-Kramer post hoc test.

We next asked whether the IL-12/STAT4 axis is involved in the formation/release of neutrophil extracellular traps (NETs) (23). First, we examined elastase release upon neutrophil activation, which occurs as part of NET formation. In contrast to an induction of neutrophil elastase release from WT cells, neither LPS nor *P*. *aeruginosa* stimulated elastase release from *Stat4^–/–^* and *Stat4^fl/fl^* *LysM^cre^* neutrophils (Figure 5A). We then further examined features of NET formation by examining cell-associated but extracellular elastase and DNA presence by flow cytometry. While LPS, heat-killed MRSA (HK-MRSA) and live MRSA, as well as Pam3CSK4 clearly induced surface-associated neutrophil elastase from WT neutrophils, STAT4 deficiency significantly attenuated surface staining of elastase (Figure 5, B and C). Importantly, the response to the Gram-positive bacteria MRSA and the Gram-negative HK-MRSA and MRSA *P*. *aeruginosa* or LPS was similarly defective in the absence of STAT4 (Figure 5, A–C). To directly test DNA release from the cells, we stained WT and STAT4-deficient neutrophils that were stimulated with live or HK-MRSA, Pam3CSK4, or LPS with Sytox Green, a cell-impermeable DNA dye. When stimulated with MRSA or Pam3CSK4, WT neutrophils showed significant externalization of DNA (Figure 5, D and E). In contrast, *Stat4^–/–^* or *Stat4^fl/fl^* *LysM^cre^* neutrophils demonstrated reduced Sytox Green signal (Figure 5, D and E). Similar results were observed with *P*. *aeruginosa* stimulation (not shown). Thus, stimulation with both Gram-positive and Gram-negative bacteria induces STAT4-dependent externalization of DNA in neutrophils, suggesting a common mechanism of response in neutrophils that involves STAT4.

**Figure 6:**
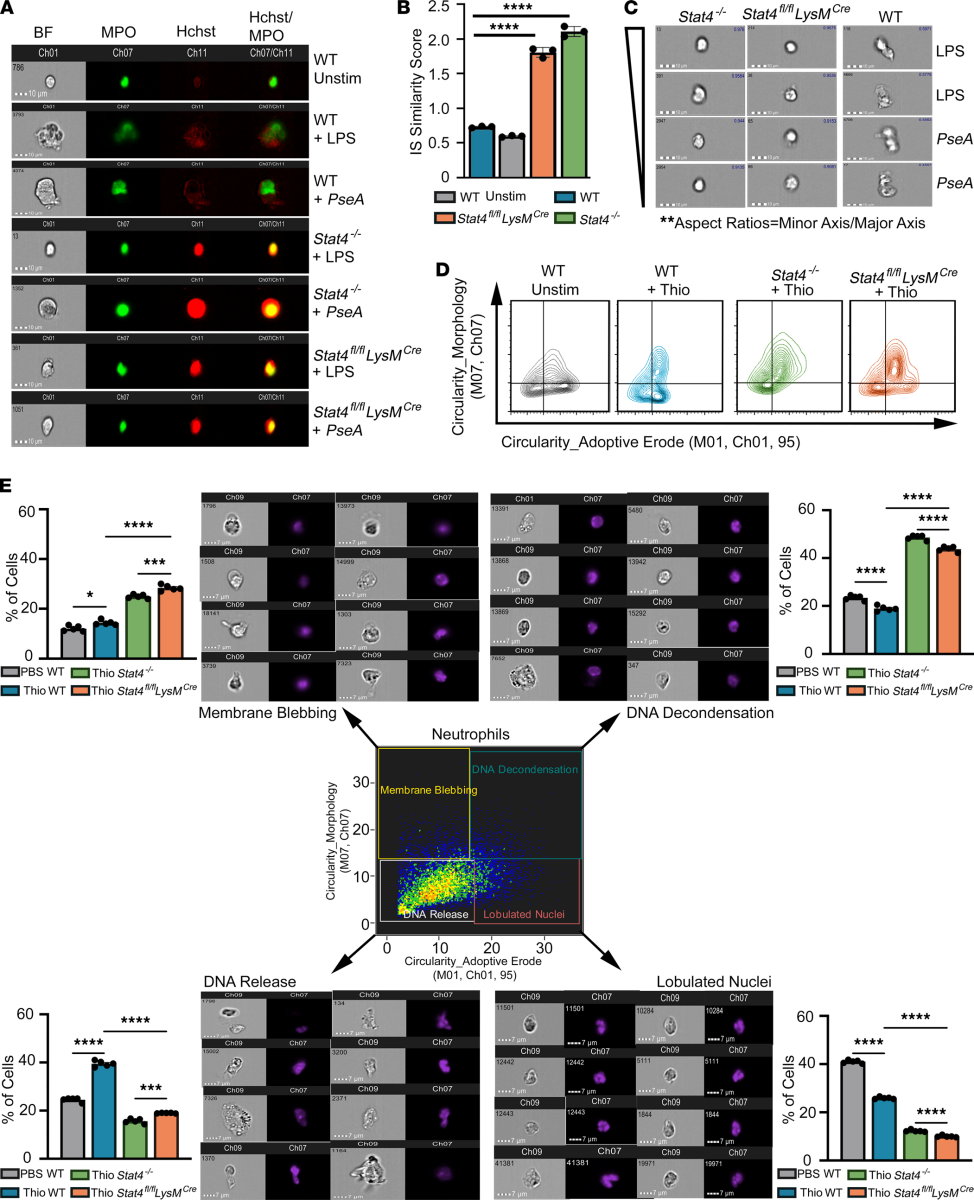
STAT4 alters multiple features of NET formation. BM neutrophils were stimulated with LPS (100 ng/mL) or *P. aeruginosa* (*PseA*; 10 MOI) and analyzed by ImageStream (IS) Multispectral Imaging Flow Cytometry. (**A**) Representative images of stimulated BM neutrophils in bright-field (BF), MPO, Hoechst (DNA staining), and overlay of MPO/Hoechst. (**B**) Colocalization of MPO and Hoechst staining (nuclear localization) calculated as a Similarity Score to quantify the degree of nuclear translocation of MPO; ***P < 0.001 using 2-way ANOVA with Tukey-Kramer post hoc test. Results are shown as the average of 50 cells for each of 3 mice in 2 independent experiments. (**C**) Morphology changes following LPS and *P*. *aeruginosa* treatment in WT, *Stat4^–/–^*, and *Stat4^fl/fl^* *LysM^cre^* neutrophils. (**D** and **E**) Singlet gated neutrophils were analyzed for nuclear circularity versus cell circularity. The images shown were obtained from events in each quadrant. (**D**) The gating strategy was applied to each of the populations of peritoneal neutrophils from WT, *Stat4^–/–^*, and *Stat4^fl/fl^* *LysM^cre^* mice 6 hours after thioglycolate injection. (**E**) Representative FACS plots and the percentage of the PC neutrophils in different stages of the NETs’ formation. *P < 0.05, ***P < 0.001, ****P < 0.0001.

Next, we applied multispectral imaging flow cytometry to examine characteristics of neutrophils undergoing “suicidal” and “vital” NETosis (24). Cells under suicidal NETosis display features of a large nuclear area, nuclear decondensation, and colocalization of myeloperoxidase (MPO) with the DNA (24). Interestingly, we detected that Hoechst-stained DNA and MPO staining were separated in WT but had overlapping signals in *Stat4^–/–^* neutrophils (Figure 6A). To quantify the colocalization of MPO and Hoechst staining, we calculated a Similarity Score (24) that reflects the degree of nuclear translocation of MPO. As shown in Figure 6B, the median Similarity Score for STAT4-deficient neutrophil was around +2.0, indicating a positive correlation between the MPO and Hoechst/DNA images and therefore increased colocalization of MPO and Hoechst nuclear staining. Thus, cell compartmentalization was altered in *Stat4^–/–^* neutrophils compared with control neutrophils as typically observed upon “suicidal” NETosis.

In contrast to “suicidal” NETosis, “vital” NETosis in neutrophils is characterized by multilobular/condensed nuclei, elongated cell shape with a polarized bleb, and minimal colocalization of MPO and nuclei (24). Upon detailed investigation of the shape and structure of WT and *Stat4^–/–^* cells, we noticed that stimulated WT cells were elongated, a change that was absent in *Stat4^–/–^* neutrophils using BF microscopy images (Figure 6C). The aspect ratio, a measure of the spherical nature of the cell, was altered in WT but not *Stat4^–/–^* cells (Figure 6C), suggesting that STAT4 deficiency reduces the signs of vital NETosis in neutrophils. We next tested NET formation in vivo using WT, *Stat4^–/–^*, or *Stat4^fl/fl^* *LysM^cre^* mice that were injected with thioglycolate. Based on the morphology/circularity parameters of cells, we analyzed 4 groups of neutrophils (Figure 6, D and E). We observed that WT, *Stat4^–/–^*, and *Stat4^fl/fl^* *LysM^cre^* neutrophils had decreased lobulated nuclei (Figure 6E), suggesting that the cells were undergoing vital NETosis (25). However, the majority of *Stat4^–/–^* cells had increased DNA condensation and diminished DNA release compared with WT cells (Figure 6E), suggesting impaired vital NETosis in the absence of STAT4.

### STAT4-deficient mice are highly susceptible to MRSA infection.

**Figure 7:**
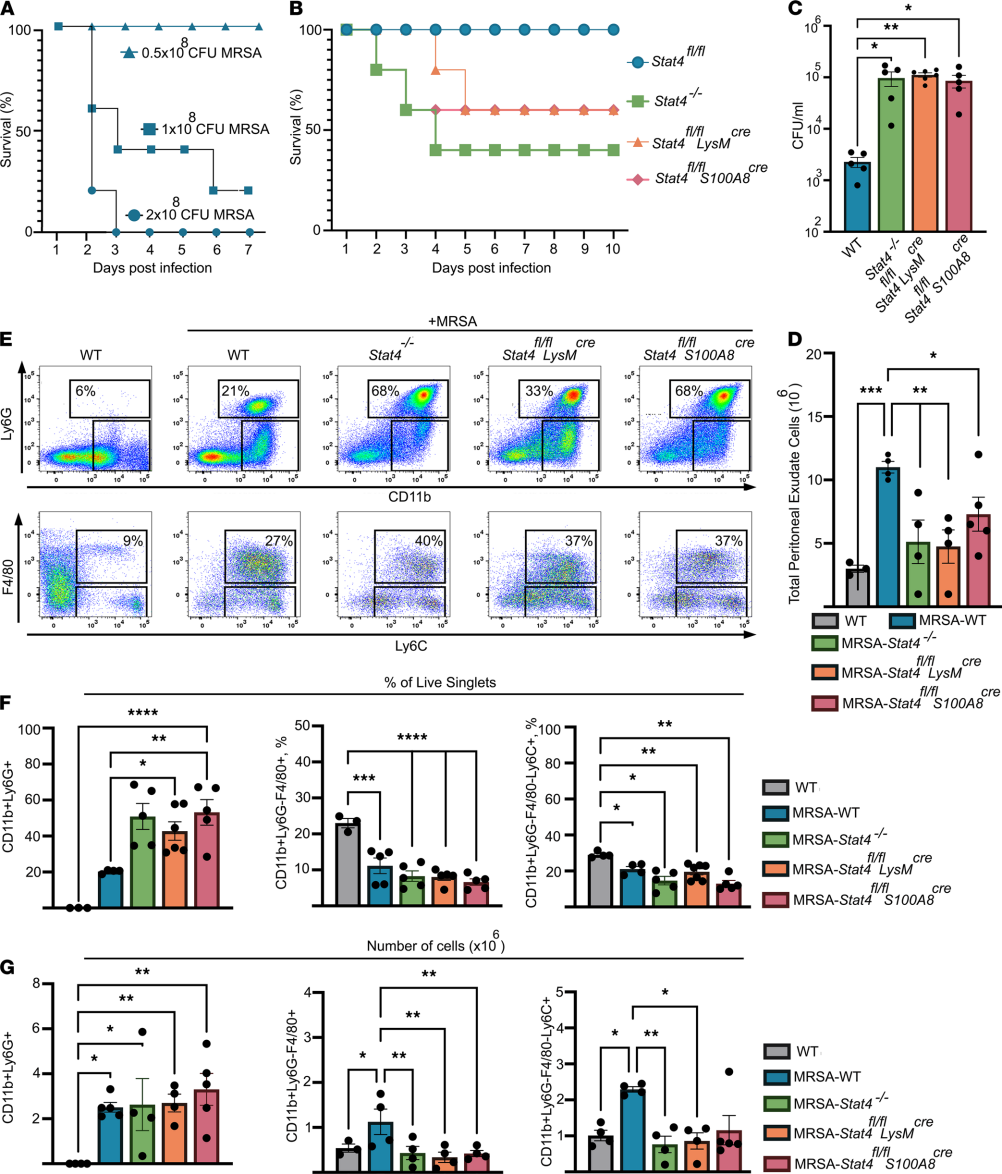
STAT4 is required for immunity to MRSA peritoneal infection. (**A**) WT mice were infected with different concentrations of MRSA. Survival rates are presented (*n* = 5–7/group). (**B**) WT (*Stat4^fl/fl^*), *Stat4^–/–^*, *Stat4^fl/fl^* *LysM^cre^*, and *Stat4^fl/fl^* *S100A8^cre^* mice were infected with MRSA (1 *×* 10^8^) i.p. Survival rates are presented (*n* = 10 mice/group). (**C**) CFU counts in the PC of WT, *Stat4^–/–^*, *Stat4^fl/fl^* *LysM^cre^*, and *Stat4^fl/fl^* *S100A8^cre^* infected mice 24 hours after MRSA infection (*n* = 5/group). (**D**) Total number of peritoneal exudate cells collected from WT, *Stat4^–/–^*, *Stat4^fl/fl^* *LysM^cre^*, and *Stat4^fl/fl^* *S100A8^cre^* mice (*n* = 4/group). (**E**) Top: Representative FACS staining of neutrophils (Ly6G^+^CD11b^+^) and bottom: myeloid subsets (Ly6C^+^F4/80^+^ and Ly6C^–^F4/80^+^ gated on CD11b^+^ events). (**F**) Total percentage and (**G**) total number of Ly6G^+^CD11b^+^ neutrophils, monocytes (Ly6C^+^F4/80^+^), and macrophages (Ly6C^–^F4/80^+^ gated on CD11b) in the peritoneal exudates of control/noninfected and MRSA-infected WT, *Stat4^–/–^*, *Stat4^fl/fl^* *LysM^cre^*, and *Stat4^fl/fl^* *S100A8^cre^* mice (*n* = 4/group). *P < 0.05, **P < 0.01, ***P < 0.001, ****P < 0.0001, using 1-way ANOVA followed by Tukey-Kramer post hoc test.

MRSA strains pose a significant public health threat in the community because they could overcome the neutrophil-mediated host defense to cause human disease (26). Because we discovered an important role of the IL-12/STAT4 axis in neutrophil functions, we next asked whether STAT4 in myeloid cells, and particularly in neutrophils, is required for immunity during MRSA infection. First, we showed that MRSA reduced the survival of WT mice in a concentration-dependent manner (Figure 7A). Next, WT (*Stat4^fl/fl^*), *Stat4^–/–^*, *Stat4^fl/fl^* *LysM^cre^*, and *Stat4^fl/fl^* *S100A8^cre^* mice were simultaneously infected with MRSA, approximately 1 *×* 10^8^ CFU, via i.p. injection. The global deletion of STAT4 resulted in increased bacterial burden and reduced survival (Figure 7, B and C). As with the experiment in global *Stat4^–/–^* mice, we observed that *Stat4^fl/fl^* *LysM^cre^* and *Stat4^fl/fl^* *S100A8^cre^* mice were highly susceptible to MRSA infection and demonstrated a substantial reduction in MRSA clearance and survival (Figure 7, B and C).

Mechanistically, MRSA infection resulted in a substantial recruitment of leukocytes to the PC, elevated percentage and number of Ly6G^+^ neutrophils, and increased number of F4/80^+^Ly6C^+^ and F4/80^+^Ly6C^–^ subsets of myeloid cells in WT (*Stat4^fl/fl^*) mice (Figure 7, E and F). These changes were accompanied by 100% survival of MRSA-infected WT mice. In contrast, reduced survival of *Stat4^–/–^*, *Stat4^fl/fl^* *LysM^cre^*, and *Stat4^fl/fl^* *S100A8^cre^* mice was associated with diminished numbers of total peritoneal cells (Figure 7D). Interestingly, the total number of F4/80^+^Ly6C^+^CD11b^+^ monocytes and F4/80^+^Ly6C^–^CD11b^+^ macrophages were reduced in *Stat4^–/–^*, *Stat4^fl/fl^* *LysM^cre^*, and *Stat4^fl/fl^* *S100A8^cre^* versus WT MRSA-infected mice, suggesting that STAT4 plays a role in the regulation of monocyte/macrophage recruitment upon MRSA infection (Figure 7, E–G). Surprisingly, STAT4 deficiency increased the relative percentage of Ly6G^+^CD11b^+^ neutrophils in the PC of MRSA-infected *Stat4^–/–^*, *Stat4^fl/fl^* *LysM^cre^*, and *Stat4^fl/fl^* *S100A8^cre^* mice in comparison with WT MRSA-infected controls (Figure 7, E–G). However, the total number of neutrophils in the peritoneum was not different between *Stat4^–/–^*, *Stat4^fl/fl^* *LysM^cre^*, *Stat4^fl/fl^* *S100A8^cre^,* and WT MRSA-infected mice. Thus, STAT4-dependent susceptibility to MRSA and inability to clear infection are likely due to defective neutrophil functions in the PC (ROS production, NETosis, bacterial killing) but not due to defective trafficking of neutrophils to the site of infection. Together, these data indicate that STAT4 is activated and required for many functions of neutrophils, thus playing a critical role in shaping antibacterial immunity.

## Discussion

This study reveals a potentially novel role for STAT4 in the regulation of neutrophil functions. While STAT4 is a well-known transcription factor for Th1 cell differentiation, its expression and role in the regulation of neutrophil functions have not been previously identified. An increasing body of evidence suggests the implication of IL-12 and STAT4 in shaping of the innate immune responses in rodents and humans (13–16, 27), but cell types that are responsible for STAT4-dependent effects have not been well defined. Here, we demonstrate that STAT4 is expressed in neutrophils and activated by IL-12 and that activation results in elevated ROS production, increased signs of NETosis, and chemotaxis. Our finding is also of high interest as these data provide evidence for a critical role of STAT4 for neutrophil-dependent immunity against bacterial infections.

While there is a significant body of knowledge about transcriptional regulation of neutrophil differentiation and maturation, much less is known about signal transduction that regulates neutrophil functions. Neutrophils quickly respond to various stimuli via engagement of cell surface receptors, including Fc, adhesion, cytokine, and chemokine receptors, as well as innate immune receptors (17). These interactions lead to an induction of intracellular signal transduction pathways that direct and orchestrate critical neutrophil functions. For example, it has been demonstrated that STAT3 regulates CXCL2-induced migration via activation of the Raf/MEK/ERK signaling axis (28). Interestingly, a role for IL-12 in myeloid cell functions became evident due to initial reports about effects of IL-12 on innate immune cells. Evidence indicates that IL-12Rβ1/2 are expressed by human eosinophils at low or undetectable levels, and PMA activation dramatically induces IL-12R expression in these cells (29). IL-12Rβ1 expression was also reported for human neutrophils (30). Here, we show that human neutrophils express both IL-12Rβ1 and IL-12Rβ2 and neutrophils are highly responsive to IL-12 stimulation in various functional assays via the activation of STAT4. As a part of an IL-12–dependent signaling cascade, IL-12 activates tyrosine kinase 2 (TYK2) and JAK2 to induce the phosphorylation of various STATs (5). JAK2 activation is required for IL-12–mediated T cell growth, whereas the TYK2/STAT4 signaling pathway is critical for IFN production. To date, little is known about a transcriptional network that regulates neutrophil functions. In this report, we demonstrated that JAK2 inhibition attenuated STAT4 phosphorylation, indicating that IL-12 activates the JAK2/STAT4 pathway in neutrophils. STATs are well-known for many functions in innate immunity. STAT1 supports IFN-dependent responses, STAT3 is downstream of IL-6, and cytokines such as colony-stimulating factors also signal via various STATs (31). We now add STAT4 to a list of transcription factors that play a crucial role in shaping of neutrophil functions.

Our data demonstrate that STAT4 deficiency has no effects on peripheral number of neutrophils under homeostatic conditions but modulates gene expression in neutrophils at the basal and activated levels. These results suggest that STAT4 activation in neutrophils is an integral component of inflammatory immunity, at least in the context of a bacterial infection. In this report, we have shown that stimulation with IL-12 activates STAT4. STAT4 might also be activated by type I IFNs and IL-23 (32), and either of those cytokines might be additional stimulators of STAT4-dependent neutrophil activity. To further explore consequences of STAT4 activation in neutrophils, we focused our attention on antimicrobial responses in neutrophils. Interestingly, we found that STAT4, at least partially, suppresses granule release from neutrophils at the basal conditions but supports activation of inflammasome and NF-κB upon the activation with IL-12. We also found that IL-12 has some STAT4-independent effects that likely work via other STATs, such as STAT1, STAT3, and STAT5. Overall, gene expression data clearly demonstrate that the IL-12/STAT4 axis actively participates in shaping neutrophil biology at homeostasis and under inflammatory conditions.

In terms of functional consequences of IL-12/STAT4-dependent activation, we first investigated ROS production by IL-12–activated neutrophils. Our data revealed that IL-12 induces oxidative burst in neutrophils and this process is STAT4 dependent. Cytokines use various signaling cascades to activate ROS production in neutrophils (33). TNF-α induces p38 MAPK–dependent mobilization of cytb558 to the plasma membrane (34). Priming of neutrophils by GM-CSF leads to the phosphorylation of p47phox and the translocation and docking of the cytosolic complex to the membrane (35). At present, the molecular basis for STAT4-dependent oxidative burst in neutrophils is not well understood, and future experiments should be focused on defining the mechanism underlying STAT4 effects in control of ROS production in neutrophils. The generation of ROS plays an important role in the antimicrobial functions of the phagocytic cells, and ROS supports phagocytosis and intracellular bacteria killing. Killing of *S. aureus* by neutrophils in vitro is highly dependent on NADPH oxidase activity (36), and our data now highlight the importance of STAT4 in this process. Importantly, STAT4 also regulates bacterial killing of MRSA that is highly associated with ROS synthesis. Phagocytosis and ROS generation are major cellular mechanisms of the microbial clearance, but their interplay is not well understood. While the main mechanism of phagocytosis is also accompanied by ROS production, recent evidence suggests that ROS-independent phagocytosis might exist (37). Our data, in line with this report, demonstrate that STAT4-dependent ROS synthesis by neutrophils has a minimal impact on successful phagocytosis of MRSA. Overall, our data indicate that effects of STAT4 are specific to certain but not all neutrophil functions.

IL-12 is induced by various types of pathogens, including bacteria, protozoa, helminths, fungi, and viruses (38). Dendritic cells, monocytes, macrophages, and to a lesser extent B cells are the source of IL-12. Additionally, microglia, infected keratinocytes and osteoblasts, and epithelial and endothelial cells serve as a source of IL-12. Neutrophils stimulated by LPS also release IL-12, and IFN-γ enhances the LPS-induced secretion of IL-12 (39). Interestingly, IL-12 is found in granules of neutrophils and is quickly released upon stimulation, suggesting that neutrophils have a positive feedback loop in their activation via IL-12. As IL-12 induces neutrophil activation and induction of the Th1 response via the induction of IFN-γ, IL-12–producing neutrophils play an active role in the regulation of innate and adaptive responses. Interestingly, we found that TLR engagement induces ROS generation in a STAT4-dependent manner. Several studies have reported that neutrophils have IL-12–containing granules that can be released upon neutrophil activation. Our data suggest that TLR-induced activation initiates IL-12 release and further supports IL-12–induced activation of neutrophils that depends on STAT4. It remains to be determined whether it could be a general mechanism for support of neutrophil functions such as bacterial killing and NETosis.

The finding that STAT4 is essential for NET formation is surprising and suggests additional STAT4-dependent immune functions of neutrophils in various disease settings. Gram-positive MRSA, an agonist to TLR2; Gram-negative bacteria *P*. *aeruginosa*; and the TLR4 agonist LPS induce elastase and extracellular DNA release that are both signs of NETosis. NETs have been shown to be important for autoimmune diseases (40), and considering the genetic link between STAT4 and various autoimmune diseases in human populations (7), it is possible that some of the impact of STAT4 on human disease is through activity in innate immune cells such as neutrophils. The details of this contribution will be important to define in future studies. NETosis is observed in 2 major forms, suicidal and vital NETosis (25). In this report, we mainly focused on the morphological changes supporting NETosis and demonstrated that STAT4 inhibits suicidal NETosis but supports steps for vital NETosis, such as changes in cell shape and DNA release. It remains to be determined how STAT4 regulates NETosis and whether activation of NADPH oxidase 2 is a necessary requirement for this process.

One of the key properties of neutrophils is an ability to migrate to a site of inflammation. Our data showed that STAT4 deficiency already diminishes chemotaxis toward CXCL1 and GM-CSF in the in vitro assays. The simplest explanation for this observation would be that STAT4 deficiency likely affects cell-intrinsic properties of neutrophils, such as motility, polarity, integrin-dependent cytoskeleton organization, or expression of chemokine receptors, and further studies should reveal a detailed mechanism for STAT4-dependent neutrophil migration.

One of the key and definitive findings of this study was demonstrating that STAT4 plays a role in antibacterial immunity via the control of MRSA bacterial load. Here, we demonstrated that global, myeloid, and neutrophil-specific STAT4 deficiency substantially reduced survival of mice after MRSA infection. Interestingly, these data also highlighted an independent role of myeloid cells, and particularly neutrophils, in this process. While peritoneal MRSA infection led to leukocyte recruitment to the peritoneum, STAT4 deficiency markedly attenuated number of peritoneal exudate cells in *Stat4^–/–^*, *Stat4^fl/fl^* *LysM^cre^*, and *Stat4^fl/fl^ S100A8^cre^* mice. In contrast to the observed defective migration in the in vitro assays, STAT4-deficient and WT neutrophils migrated equally well to the site of infection. The detected differences between the in vitro Transwell assays and in vivo observed phenotypes might be due to a more complex milieu of signals that are involved in neutrophil recruitment in vivo. Peritoneal cell exudate from WT-infected mice also contained F4/80^+^Ly6C^+^ monocytes and F4/80^+^Ly6C^–^ macrophages, and these populations were reduced in *Stat4^–/–^* and *Stat4^fl/fl^* *LysM^cre^*, as well as *Stat4^fl/fl^ S100A8^cre^* mice likely due to indirect effects of STAT4-deficient neutrophils on monocyte recruitment to the peritoneum at least in the case of *Stat4^fl/fl^* *S100A8^cre^* mice. If the numbers of neutrophils are similar in the peritoneum between STAT4-deficient and WT mice, why do *Stat4^–/–^*, *Stat4^fl/fl^* *LysM^cre^*, and *Stat4^fl/fl^* *S100A8^cre^* mice show increased MRSA burden and reduced survival? Neutrophil binding and ingestion of invading *S*. *aureus* trigger potent oxidative and nonoxidative antimicrobial killing mechanisms (41). We believe while STAT4-deficient neutrophils were able to migrate to the infected peritoneum, their flawed capacity to produce ROS and NETs and perform bacterial killing was a critical component that is lacking in the successful elimination of MRSA infection.

In summary, in this report, we described a requirement for STAT4 in regulation of several key neutrophil functions and demonstrated that STAT4 plays a critical role in antimicrobial immunity. We have used a newly generated myeloid cell–specific and neutrophil-specific STAT4-deficient mouse model that allowed us to exclude any additional effects from other STAT4-expressing cells. The study reported here might also raise some questions about interpretations of previous work involving STAT4. While much of the function of STAT4 has been attributed to T cells, the results from in vivo studies using germline *Stat4^–/–^* mice could be reexamined from the perspective that STAT4 could contribute to the activity of innate immune cells that might also affect disease phenotypes. Defining exactly how the IL-12/STAT4 axis controls neutrophil activation should be an interesting and important area of further investigation.

## Methods

### Mice.

The study was performed with C57BL/6J (WT) (The Jackson Laboratory, JAX: 000664) mice, mice that have germline deletion of Stat4 (*Stat4^–/–^*), and the conditional mutant allele in *Stat4: Stat4^fl/fl^* *LysM^cre^* and *Stat4^fl/fl^* *S100A8^cre^* mice. *Stat4^fl/fl^* mice were generated by Ozgene Inc. (Australia), and breeding pairs were shipped to Eastern Virginia Medical School and Indiana University to generate *Stat4^fl/fl^* *LysM^cre^* and *Stat4^fl/fl^* *S100A8^cre^* mice. Breeding pairs of *LysM^cre^* and *S100A8^cre^* (JAX: 004781 and JAX: 021614, respectively) were purchased from JAX. All mice were on C57BL/6 background. Mice were generated with the third exon in the *Stat4* gene being flanked with *loxP* sites and containing an FRT-flanked neogene. Correctly targeted pups were identified by Southern blot and crossed to Flp-transgenic mice to delete the neogene. Neodeleted *Stat4^+/fl^* mice were crossed with the specified Cre-transgenic mice to mediate deletion of exon 3 in a lineage-specific manner. This approach was chosen since splicing of the remaining exons (with the ATG start codon in exon 2) will result in a nonsense transcript arising from a frameshift during translation.

### Western blotting.

BM neutrophils from WT, *Stat4^fl/fl^* *LysM^cre^*, and *Stat4^fl/fl^* *S100A8^cre^* mice were isolated via negative selection kit (STEMCELL Technologies, 19762A) and stimulated with 40 ng/mL IL-12 (Peprotech, 210-12) in RPMI with 10% FBS for the indicated time points. Following stimulation, cells were washed and lysed with lysis buffer (Thermo Fisher Scientific, 78501) contacting protease inhibitor (Thermo Fisher Scientific, 78429) and phosphatase inhibitor cocktails (Cell Signaling Technology, 5870S). Protein content was determined by BCA method (Thermo Fisher Scientific), and 20 μg of protein lysate was resolved on a 4%–20% polyacrylamide gradient gel, followed by transfer to PVDF membrane via semidry transfer. Membranes were blocked at room temperature for 1 hour with TBS-based blocking buffer (LI-COR Biosciences 927-50000). The following primary Abs were incubated at 4°C overnight: STAT4 (Cell Signaling Technology C46B10), p-STAT4 (BD Biosciences 612739), JAK2 (Cell Signaling Technology 3230), p-JAK2 (Cell Signaling Technology, 3771), and GAPDH (Santa Cruz Biotechnology, 32233). Secondary Abs (926-68072 and 926-32211, Li-COR Biosciences) were incubated at room temperature for 1 hour. Images were acquired using the LI-COR Odyssey system. Band intensity was determined using Image Studio Lite software (LI-COR Biosciences).

### Antimicrobial pathway PCR arrays.

Isolated peripheral blood neutrophils (2 × 10^6^) from WT and *Stat4^–/–^* mice were incubated with or without IL-12 (100 ng/mL) for 3 hours in complete RPMI-1640 medium (Gibco, Thermo Fisher Scientific). Total RNA from all groups was extracted using RNeasy Mini Kit (QIAGEN, 74106). The concentration and purity of the RNA were determined using a NanoDrop 8000 spectrophotometer (Thermo Fisher Scientific). To eliminate the possible amplification of contaminating genomic DNA, DNase treatment (QIAGEN) was carried out, and total RNA (1 μg) was reverse-transcribed to cDNA using the RT2 First Strand Kit (QIAGEN, 330404). Mouse RT² Profiler PCR Array Mouse Antibacterial Response (PAMM-148Z, QIAGEN) was used for the analysis. A negative control for genomic DNA and contaminating RNA was also conducted in each sample. Amplification, data acquisition, and the melting curve were carried out by the Real Time PCR system (Applied Biosystems, Thermo Fisher Scientific). The Ct and melting curve of each gene were automatically established and recorded by the software. The ΔCt method was used for PCR array data analysis. The normalized ΔCt for each gene of interest (GOI) was calculated by deducting the average Ct of the 5 housekeeping genes from the Ct of each GOI. Then the double delta Ct (ΔΔCt) for each GOI was calculated by deducting the average ΔCt of GOI in the sham group from the ΔCt of each GOI. The fold change of each GOI compared with the sham group was calculated as 2^-ΔΔCt^.

### Murine and human neutrophil isolation.

BM neutrophils were isolated from the PBS-flushed femur and tibia bones of BM of 6- to 12-week-old mice. Obtained cell suspension was washed, then resuspended at 1 × 10^8^ cells/mL in PBS + 0.5% BSA and 2 mM EDTA. Murine neutrophils were isolated from the peripheral blood or BM using EasySep Mouse Neutrophil Enrichment Kit (STEMCELL Technologies), and human neutrophils were isolated from peripheral blood of healthy donors at Eastern Virginia Medical School using EasySep Direct Neutrophil Isolation Kit (STEMCELL Technologies).

### Transwell migration assays.

BM neutrophils (0.25 × 10^6^) from WT, *Stat4^–/–^*, and *Stat4^fl/fl^* *LysM^cre^* mice were suspended in 0.1 mL RPMI 1640 supplemented with 1% FBS and 10 mM HEPES (“migration media” hereafter) and seeded onto the top well of the Transwell. The bottom wells were loaded with 0.6 mL migration media supplemented with the following: media alone, 12–100 ng/mL rmCXCL1, or 50 ng/mL rGM-CSF (both Peprotech). The loaded cells were allowed to migrate for 3 hours at 37°C before being harvested and counted. The migration index indicates the percentage of transmigrated neutrophils normalized to the mean of the controls for each genotype.

Isolated human peripheral blood neutrophils were resuspended in migration media and incubated for 30 minutes with IL-12 (40 ng/mL) before loading into Transwell inserts (3.0 μm pore, Corning). The top wells of the Transwell chambers were loaded with 0.5 *×* 10^6^ neutrophils in 200 μL of migration media. The bottom wells were loaded with 0.6 mL migration media either alone or supplemented with 100 ng/mL rhCXCL1 or 50 ng/mL rGM-CSF (both Peprotech). Neutrophils were allowed to migrate for 90 minutes at 37°C before being harvested and counted. The migration index indicates the percentage of transmigrated neutrophils normalized to the mean of the controls for each condition.

### CXCL1 and GM-CSF–induced recruitment of neutrophils.

WT, *Stat4^–/–^*, and *Stat4^fl/fl^* *LysM^cre^* mice were injected i.p. with either 300 ng rmCXCL1, 300 ng rmGM-CSF, or 0.9% NaCl for negative control. After 4 hours, mice were sacrificed; peritoneal lavage was collected, stained for CD11b^+^Gr-1^+^ cells, and analyzed by FACS.

### Flow cytometry.

Single-cell suspensions from the BM, peritoneal cell exudate, and heparinized peripheral blood were stained with Abs as we previously described (42) and analyzed using a Cytek DXP8 Color (Cytek Development Inc.) with upgraded FACSCalibur (BD Biosciences) for ROS production, p-STAT4, IL-12Rβ1/2, and purity check staining and Attune NxT flow cytometer (ABI) for elastase and Sytox Green staining. Data were analyzed using FlowJo (Tree Star Inc.). For all flow cytometry experiments, gates were set based on isotype and fluorescence minus one controls. The anti-mouse Abs used were as follows: Ly6C-APC (HK1.4), CD45-PerCP (30-F11), CD11b–Pacific Blue (M1/70.15), Ly6G-FITC (1A8), F4/80-eFluor450 (BM8.1), and anti-mouse CD16/CD32 Abs (Lymphocyte Culture Center, University of Virginia Health System) for blocking. For FACS on human neutrophils, 100 μL whole blood was stimulated with 40 ng/mL rhIL-12 for 0, 15, or 60 minutes. Following IL-12 stimulation, cells were surface stained with anti–CD15–Pacific Blue (clone MMA, BioLegend), anti–CD16-PerCPCy5.5 (clone 3G8, BioLegend), and anti–IL-12Rβ2–Alexa Fluor 488 (clone 305719, R&D Systems, Bio-Techne) or anti–IL-12Rβ1–FITC (clone 69310, R&D Systems, Bio-Techne) for 30 minutes at 4°C. Following surface staining, cells were fixed and permeabilized for intracellular p-STAT4 detection using the True-Nuclear Transcription Factor Buffer Set (BioLegend), following the manufacturer’s protocol. Cells were incubated with anti–p-STAT4–PE Abs (clone 4LURPIE, BioLegend) for 30 minutes at 4°C. To distinguish between live and dead cells in flow cytometry experiments, a LIVE/DEAD Aqua Dead Cell Stain Kit (Invitrogen, Thermo Fisher Scientific) was used.

### Detection of ROS.

BM was harvested from 6- to 10-week-old WT, *Stat4^fl/fl^* *LysM^cre^*, or *Stat4^fl/fl^* *S100A8^cre^* mice by flushing the femur and tibia bones with cold PBS. Cells were centrifuged at 300*g* for 7 minutes and resuspended in 500 μL advanced RPMI (Gibco, Thermo Fisher Scientific). Neutrophils were stained with 0.2 μM DCFDA (Thermo Fisher Scientific, D-399) for 30 minutes at 37°C in the dark. Next, cells were incubated with either 40 ng/mL IL-12 (Peprotech, 210-12) or LPS (100 ng/mL) in advanced RPMI at 37°C with 5% CO_2_. In some experiments for LPS-induced ROS production, neutrophils were pretreated with IL-12 neutralizing Ab (C17.8, BioLegend). After 1 or 14 hours, neutrophils were collected, washed with 1× wash buffer, stained with anti-CD11b and anti-Ly6G Abs, and analyzed by FACS.

### Apoptosis.

BM was harvested from 6- to 10-week-old WT mice and incubated with 40 ng/mL IL-12 (Peprotech, 210-12) for 30 minutes, 2 hours, 6 hours, and 12 hours at 37°C with 5% CO_2_. Following IL-12 incubation, 1 *×* 10^6^ cells were washed twice with ice-cold PBS, resuspended with 1 mL of binding buffer (0.01 M HEPES at pH 7.4, 0.14 M NaCl, 2.5 mM CaCl_2_), stained with annexin V–APC and 7-amino-actinomycin D (BD Pharmingen, 550475 and 559925, respectively) for 15 minutes at 25°C in the dark, washed, and analyzed by FACS within 1 hour.

### Phagocytosis and killing assays.

Bacterial phagocytosis and killing were performed as previously described (43). Briefly, WT or STAT4-deficient neutrophils (2 *×* 10^5^/well) were plated into 2 individual 96-well plates with opaque walls and clear bottoms. Cells were pretreated with IL-12 for 1 hour before the addition of GFP-MRSA at a multiplicity of infection of 50:1. Infected cells were incubated 1 hour to allow phagocytosis, both plates were washed with warm PBS, and GFP fluorescence was measured on the first plate. The second plate was then maintained in PBS with or without IL-12 and was incubated for another 2 hours for killing assays. To measure the intensity of intracellular GFP fluorescence, extracellular fluorescence was quenched with 500 μg/mL trypan blue, and the GFP fluorescence was quantified using a fluorimeter plate reader (SpectraMaxGemini EM Fluorometer, 485 nm excitation/535 nm emission, MolecularDevices). Trypan blue served as a blank. A reduction in GFP fluorescence in the killing plate relative to the phagocytosis plate indicated bacterial killing. To examine phagocytosis for *E. coli*, the Vybrant Phagocytosis Assay Kit (Thermo Fisher Scientific V-6694) was performed following the manufacturer’s instructions. Briefly, WT or STAT4-deficient BM neutrophils were incubated with fluorescein-labeled *E*. *coli* (K-12 strain) BioParticles (Thermo Fisher Scientific) in complete RPMI media for the indicated time points. Following several wash steps, any membrane-bound, nonphagocytosed bioparticles were quenched with trypan blue. Fluorescein-positive cells were then detected and quantified by FACS.

### Neutrophil elastase and Sytox Green assays.

Murine neutrophils isolated from BM were stimulated with LPS (100 ng/mL) or *P. aeruginosa* (10 MOI). Supernatant from each condition was assayed for neutrophil elastase using the Neutrophil Elastase/Ela2 ELISA kit (DY4517-05, R&D Systems, Bio-Techne). Alternatively, BM neutrophils were isolated from BM and stimulated with LPS (100 ng/mL), MRSA (10:1 MOI), HK-MRSA (10:1 MOI equivalent), or Pam3CSK4 (10 ng/mL) for 60 minutes. Cell-associated neutrophil elastase and DNA were determined by staining with specific Ab or Sytox Green (Thermo Fisher Scientific, S7020), respectively, and analyzed by FACS.

### Imaging flow cytometry.

We applied multispectral imaging flow cytometry to examine characteristics of neutrophils undergoing suicidal and vital NETosis as described (24). Briefly, ex vivo–stimulated BM neutrophils or peritoneum neutrophils from the thioglycolate peritonitis model were tested for changes in cell morphology during NETosis. One million ex vivo neutrophils were stimulated with 1 μg/mL of LPS or *P. aeruginosa* (10 MOI) for at 37°C with 5% CO_2_. After 1 hour, neutrophils were centrifuged at 500 RCF for 5 minutes at room temperature and resuspended in 100 μL of 2% paraformaldehyde containing 1:1000 diluted Hoechst prior to the acquisition on ImageStream Multispectral Imaging Flow Cytometer (Luminex). For MPO staining, neutrophils were incubated with 0.2% donkey serum (MilliporeSigma) in PBS for 30 minutes, then incubated with anti-mouse MPO Abs (clone 2D4, Abcam), diluted in blocking buffer for 1 hour at 37°C, and washed with PBS. Images were acquired on the ImageStream Multispectral Imaging Flow Cytometer, using the 40× and 60× magnification objective, which provides a numerical aperture of 0.9 and a pixel dimension of 0.3 m × 0.3 m. A core diameter of 7 μm was used in order to minimize in-focus events. The 405 nm excitation laser was used at an output power of 1 to 10 mW depending on the intensity of staining. In order to avoid debris and cell aggregate effects, only objects with a minimum cross-sectional area of 50 m^2^ and a maximum of 600 m^2^ were collected. Finally, we set up the number of acquiring images to 50,000 cells. All analysis was based on a subsequent report (24). For cell analysis, cell imagery was analyzed using the requisite Image Data Exploration and Analysis Software, version 6.1 (Amnis Corporation). The best focused cells were selected using BF Gradient RMS, a measurement of image contrast that excludes out-of-focus events (area vs. Gradient RMS). Doublets were excluded using SSC Intensity/Hoechst, and only cells with a spot distance of 0–1 (cells with either single nuclear signal or an elongated signal) were included in the analysis. A Similarity Score that reflects the degree of nuclear translocation of MPO was calculated as described (24). The selected neutrophils were also analyzed for nuclear circularity (nuclear stain, *y* axis, labeled as Circularity_Morphology: M07, Ch07) versus cell circularity (BF, *x* axis, labeled as Circularity_Adaptive Erode: M01, Ch01, 95), and 4 different quadrants are representative of each stage of NETosis as identified by changes in the cellular and nuclear morphologies.

### Thioglycolate peritonitis model and Sytox Green flow cytometry.

WT, *Stat4^–/–^*, and *Stat4^fl/fl^* *LysM^cre^* mice were injected with 3% of thioglycolate broth (Thermo Fisher Scientific) or PBS as a control i.p. to elicit a robust influx of neutrophils into the PC. After 6–72 hours, the peritoneal lavage was collected to investigate the morphological changes and immune responses of neutrophils. A cell-impermeable, DNA-binding Sytox Green dye (Thermo Fisher Scientific, S7020) was used for measuring the NET release. After filtering out the debris with a mesh, neutrophils were analyzed using Attune flow cytometer (Applied Biosystems, Thermo Fisher Scientific). Because Sytox Green expresses fluorescence only after binding to DNA, the step to remove unbound dye can be omitted. FSC and SSC were used to select mainly neutrophils.

### MRSA-induced peritoneal infection.

The methicillin-resistant *S. aureus* USA 300 lac strain stocks (provided by Bethany Moore, University of Michigan) were stored at –80°C and cultivated in tryptic soy broth medium (Thermo Fisher Scientific). WT, *Stat4^–/–^*, *Stat4^fl/fl^* *LysM^cre^*, and *Stat4^fl/fl^* *S100A8^cre^* mice were infected i.p. with approximately 1 × 10^8^ CFU of MRSA in 200 μL of PBS, and animal survival was monitored until day 10. For the acute infection study, WT, *Stat4^–/–^*, *Stat4^fl/fl^* *LysM^cre^*, and *Stat4^fl/fl^* *S100A8^cre^* mice were infected as above and euthanized 16 hours after infection, and peritoneal lavage and the blood were collected to measure bacteria load and analyze peritoneal cell exudate in the recipients using FACS.

### Determination of bacterial load.

Peritoneal lavage (dilution 1:100/PBS) and blood (no dilution) from WT and *Stat4^–/–^*, *Stat4^fl/fl^* *LysM^cre^*, and *Stat4^fl/fl^* *S100A8^cre^* were used to determine bacterial counts. The dilutions were plated on tryptic soy agar medium (Thermo Fisher Scientific), and colonies were incubated at 37°C, 5% CO_2_, for 18 hours followed by CFU count. Bacterial burdens were normalized to volume of peritoneal lavage (3 mL/cavity) and calculated by the following equation: (CFU/mL plated) × (dilution factor)/volume mL. CFU/mL represents bacterial burdens in the fluids.

### Isolation of BMDMs.

BM was harvested from 6- to 10-week-old mice by flushing the femur and tibia bones using cold PBS supplemented with 0.5% BSA and 2 mM EDTA. Cells were centrifuged at 500*g* for 10 minutes at room temperature. The supernatants were discarded and cell pellets were resuspended in DMEM F12-10 medium, which was supplemented with 10 mM l-glutamine and 100 U/mL recombinant M-CSF. A total of 2.5 *×* 10^5^ cells/mL were then cultured in 10 mL sterile plastic Petri dishes and incubated in a 37°C, 5% CO_2_, incubator for 3 days and then nourished more with the supplemental DMEM F12-10 until day 7.

### Statistics.

For comparisons between 2 groups, unpaired or paired 2-tailed Student’s *t* test was used. Normality was determined using Shapiro-Wilk testing. For comparisons of more than 2 conditions with a defined control group (Transwell experiments), a 1-way ANOVA with post hoc test was used. For all experiments, the means ± SEM are shown. Significant differences were defined as *P* < 0.05.

### Study approval.

Mice were maintained under specific pathogen–free conditions at Indiana University, Vanderbilt University, and Eastern Virginia Medical School. All experiments were performed with the approval of the IACUCs of Indiana University, Vanderbilt University, and Eastern Virginia Medical School. All aspects of animal research and husbandry were conducted in accordance with the federal Animal Welfare Act and the NIH’s *Guide for the Care and Use of Laboratory Animals* (National Academies Press, 2011). Collection of human peripheral blood was performed with written informed consent and approved by the IRB of Eastern Virginia Medical School.

## Author contributions

EVG, JLN, CHS, and MHK conceived and designed the study. PMB, AKM, WCK, ASN, MH, and MSB conducted experiments. PMB, AKM, PDM, WCK, NSA, ASN, MH, MSB, JLN, CHS, MHK, and EVG contributed to data analysis. EVG and MHK wrote the manuscript, and PMB, AKM, PDM, WCK, NSA, ASN, MH, MSB, JLN, CHS, MHK, and EVG contributed to the manuscript editing.

## Supplementary Material

Supplemental data

## Version 1 06/17/2021

In-Press Preview

## Version 2 07/22/2021

Electronic publication
