# Supplementary material for: STAT4 is expressed in neutrophils and promotes antimicrobial immunity
Source: JCI Insight. 2021 Jul 22;6(14):e141326. doi: 10.1172/jci.insight.141326 (PMC8410094; doi:10.1172/jci.insight.141326)
Supplement: Supplemental data [file jciinsight-6-141326-s140.pdf]

Supplemental Materials

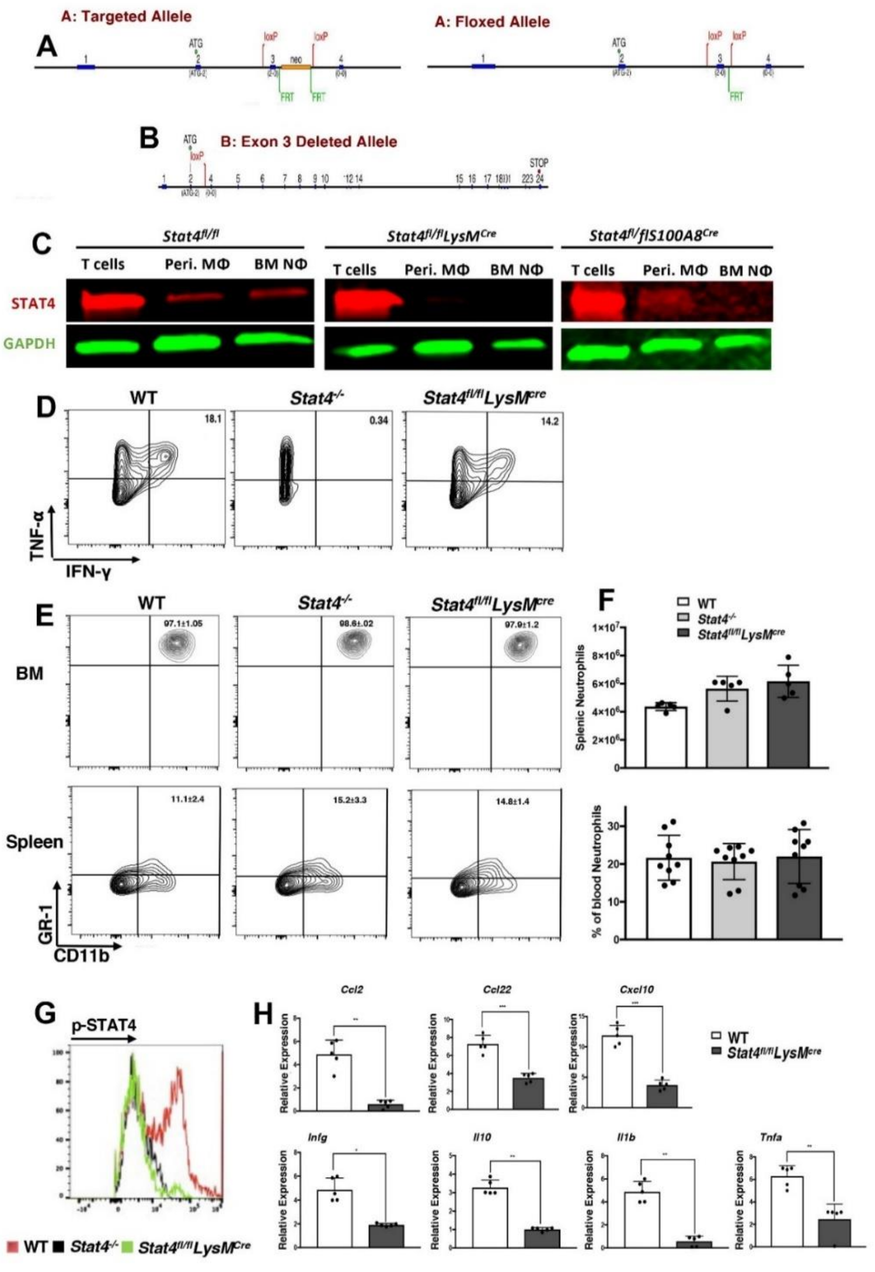

Supplemental Figure 1

Supplemental Figure 1: Development of *Stat4*$^{fl/fl}$*LysM*^{cre}, and *Stat4*$^{fl/fl}$*S100A8*^{cre} mice. **(A)** Schematic map of the *Stat4* locus showing the inserted loxP sites flanking the third exon and the FRT-flanked neo cassette before (top) and after (bottom) Flp-mediated deletion. **(B)** Schematic of the targeted Stat4 locus following Cre-mediated deletion. The third exon was chosen for targeting because removal causes a nonsense frameshift following splicing of the remaining exons. **(C)** STAT4 expression in T cells, peritoneal macrophages, and BM neutrophils isolated from WT, *Stat4*$^{fl/fl}$*LysM*^{cre}, and *Stat4*$^{fl/fl}$*S100A8*^{cre} mice. **(D)** Naïve CD4 T cells from WT, *Stat4*$^{-/-}$, and *Stat4*$^{fl/fl}$*LysM*^{cre} mice were differentiated under Th1 conditions for 5 days, then activated with PMA/ionomycin for 6 hours, and stained with intracellular cytokine Abs. **(E)** Representative FACS plots for CD11b^{+}Gr-1^{+} neutrophils in the BM and the spleen of WT, *Stat4*$^{-/-}$, and *Stat4*$^{fl/fl}$*LysM*^{cre} mice. **(F)** Number of neutrophils in the spleen and peritoneal blood of WT, *Stat4*$^{-/-}$, and *Stat4*$^{fl/fl}$*LysM*^{cre} mice. **(G-H)** Bone marrow derived macrophages (BMDM) from WT, *Stat4*$^{-/-}$, and *Stat4*$^{fl/fl}$*LysM*^{cre} mice were activated with HK-MRSA and IL-12. (**G**) Representative FACS plot for pSTAT4. **(H)** Cytokine and chemokine levels by qPCR. Data are average±SEM. * p<0.05, **p<0.01.

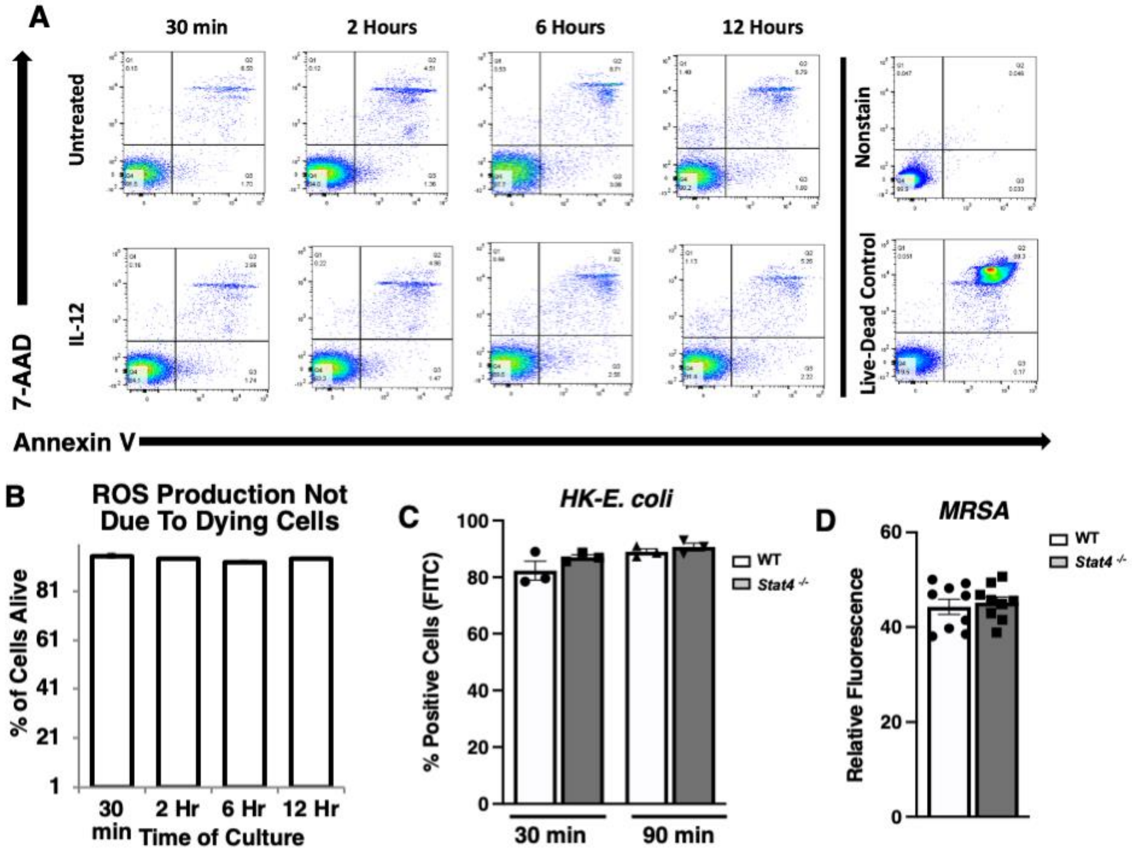

Supplemental Figure 2. STAT4-deficiency has no impact on neutrophil apoptosis and phagocytosis. **(A-B)** Neutrophils were incubated with or without IL12 (40 ng/ml) in complete RPMI media. After different time points (0.5-12 hrs), neutrophil were collected, stained with Annexin V and 7-AAD, and analyzed by FACS (n=2/per group). Representative FACS plot and averages for live cells are shown. **(C-D)** WT and *Stat4*$^{-/-}$ bone marrow neutrophils were incubated with fluorescein-labeled K-12 *E. coli or MRSA.* After 30 and 90 min, neutrophils were collected, washed, non-phagocytosed bioparticles were quenched with trypan blue, and fluorescein positive cells were detected using FACS. Data are average±SEM.
